# Effects of probiotics in patients with morbid obesity undergoing bariatric surgery: a systematic review and meta-analysis

**DOI:** 10.1038/s41366-023-01375-5

**Published:** 2023-09-06

**Authors:** Yuting Wang, Youwei Zheng, Lirun Kuang, Keyu Yang, Jiaji Xie, Xinde Liu, Shan Shen, Xinchao Li, Shiran Wu, Yuyi Yang, Jiafei Shi, Jialiang Wu, Yong Wang

**Affiliations:** Department of General Surgery, The Fourth Affiliated Hospital of China Medical University, Shenyang, 110032 China

**Keywords:** Weight management, Obesity, Bariatric surgery, Obesity

## Abstract

**Background:**

Probiotics are commonly used after bariatric surgery. However, uncertainty remains regarding their effects. The purpose of this systematic review was to assess the effect of probiotics in patients with morbid obesity undergoing bariatric surgery.

**Methods:**

PubMed, Cochrane Library, Embase, Science Direct, and Web of Science were searched from inception to April 4, 2023. No language restrictions were applied. Relevant randomized controlled trials and controlled clinical trials were included. We used the aggregated data extracted from the trials and assessed the heterogeneity. When severe heterogeneity was detected, a random effect model was used. All stages of the review were done by independent authors.

**Results:**

We screened 2024 references and included 11 randomized controlled trials and controlled clinical trials. Compared with the protocol groups, probiotics showed significant effects on regulating aspartate amino transferase level (MD = −4.32 U/L; 95% CI [−7.10, −1.53], *p* = 0.002), triglycerides (MD = −20.16 mg/dL; 95% CI [−34.51, −5.82], *p* = 0.006), weight (MD = −1.99 kg; 95% CI [−3.97, −0.01], *p* = 0.05), vitamin B_12_ (MD = 2.24 pg/dL; 95% CI [−0.02, 4.51], *p* = 0.05), dietary energy (MD = −151.03 kcal; 95% CI [−215.68, −86.37], *p* < 0.00001), dietary protein (MD = −4.48 g/day, 95% CI [−8.76, −0.20], *p* = 0.04), dietary carbohydrate (MD = −34.25 g/day, 95% CI [−44.87, −23.62], *p* < 0.00001), and dietary fiber (MD = −2.17 g/day, 95% CI [−3.21, −1.14], *p* < 0.0001). There were no severe side effects related to probiotics.

**Conclusions:**

Our meta-analysis suggested that probiotics may delay the progression of liver function injury, improve lipid metabolism, reduce weight, and reduce food intake, although the effects on other indicators were insignificant. Probiotics may be helpful for patients undergoing bariatric surgery. The review was registered on PROSPERO (International prospective register of systematic reviews): CRD42023407970. No primary source of funding.

## Introduction

Obesity is a multifactorial disease that accumulates excess body fat and leads to negative health effects [1]. Over the past 40 years, the global prevalence of obesity has increased dramatically, from 3% to 11% in men and from 6% to 15% in women over the same period [2]. Moreover, compared to normal weight, obesity is associated with significantly higher all-cause mortality [3, 4]. With social lifestyles change, the number of patients with obesity is increasing constantly, placing a serious burden on public health [4, 5].

Obesity remains a largely refractory disease to dietary and pharmacological treatment, but responds well to bariatric surgery generally [6]. Bariatric surgery was reported to result in significant weight loss and may induce remission or improvement in obesity-related risks and complications [7, 8].

In recent years, the field of probiotics has been booming. Probiotics are active microorganisms that are beneficial to the host people. Obesity is associated with reduced gut microbial diversity and high rates of micronutrient deficiency [9]. What’s more, oral probiotics can modulate the structure of intestinal microbiome and the altered gut microbiome may influence inflammatory pathways, glucose and lipid metabolism in the host [10–12]. In addition, alterations in the gut microbiome were shown to affect these host responses in other settings [13, 14]. Therefore, probiotics were suggested as a therapeutic strategy in patients with obesity for being effective in reducing body mass index and waist circumference [1].

An extensive systematic review on the effects of probiotics on patients with morbid obesity undergoing bariatric surgery has not been conducted. This systematic review and meta-analyses of RCTs aimed to extensively assess the effects of probiotics supplementation in patients with morbid obesity undergoing bariatric surgery.

## Methods

A protocol of the study had been registered in PROSPERO database of systematic review protocols on March 26, 2023, with identification number CRD42023407970.

### Searches and selection strategy

We searched electronic databases of PubMed, Cochrane Library, Embase, Science Direct and Web of Science, from inception to March 14, 2023, using a combination of subject terms and free words. There were no language or date restrictions. Search terms included: probiotics, probiotic, probiotic*, prebiotics, prebiotic, prebiotic*, synbiotics, synbiotic, synbiotic*, randomized controlled trial, bariatric surgery, RYGB (Roux-en-Y gastric bypass), LSG (Laparoscopic Sleeve Gastrectomy), SG (Sleeve Gastrectomy), OAGB (One-Anastomosis Gastric Bypass). Additionally, references of included studies as well as any systematic review, meta-analysis, and practice guideline relevant to the topic were checked manually to identify studies that were not captured by the online electronic searches. All of the above work was done by independent researchers and was approved by third reviewers.

### Inclusion criteria

Studies were selected for inclusion by two independent reviewers and the work was approved by third reviewers. Here are the inclusion criteria: (A) The participants were adults (≥18 years) with morbid obesity (BMI ≥ 40 or as BMI ≥ 35 with accompanying obesity related co-morbidities such as type 2 diabetes, hypertension, obstructive sleep apnea, and others [15]) who received any kind of bariatric surgery (B)The patients were subjected to probiotics at any dose and for any duration. Probiotics were defined as “living microorganisms which when administered in adequate amounts confer a health benefit on the host [16]”. The patients did not use any antibiotics prior to the beginning of the study. (C) The study design was a randomized controlled clinical trial (RCT) or a controlled clinical trial (CCT). (D) The study compared any type of probiotics or synbiotics (a combination of probiotics and prebiotics) with placebo, digestive enzymes, care as usual, and no intervention.

### Exclusion criteria

Studies that included patients who had undergone any other gastrointestinal procedures were excluded. Studies comparing probiotics with other interventions rather than placebo were also excluded, as they would also affect the results.

### Screenings and data extraction

Identified references were checked for duplications using Endnote software. Screening of titles, abstracts, and full texts was also done by using Endnote. After meeting the inclusion and exclusion criteria, the included studies were reviewed using a standardized template. The subsequent data were extracted: (A) basic features: author, publication year, study design, number of participants, intervention, and outcomes. (B) methods: randomization, allocation concealment, blindness, data integrity, selective reporting, and other biases; (C) intervention measures: specific medication, dose, treatment duration; (D) outcome biomarkers: liver function: serum ALT (Alanine Aminotransferase), AST (Aspartate Aminotransferase), GGT (Glutamyl Transpeptidase); glycemic parameters: plasma glucose, insulin, HbA1c (Hemoglobin A1c), HOMA-IR (Homeostatic Model Assessment for Insulin), QUICKI(Quantitative Insulin Sensitivity Check Index); blood lipid levels: TC (Total Plasma Cholesterol), TG (Triglyceride), HDL (High-Density Lipoprotein), LDL (Low-Density Lipoprotein); inflammatory factor levels: serum IL-6 (Interleukin-6)levels, TNF-a (Tumor Necrosis Factor-a)levels, CRP (C-reactive protein); general measures: %EWL (%Excess Weight Loss), BMI (Body Mass Index), weight, waist circumstance (WC); serum vitamin B_12_, 25-hydroxy vitamin D_3_, folate; food intake: dietary energy, dietary protein, dietary cholesterol, dietary fat, dietary fiber; ferritin, Hb (Hemoglobin), BES (Binge Eating Score), YFBS (The Yale Food Addiction Scale), GSRS (Gastric Symptom Rating Scale), and adverse events. Both phases were preceded by pilot screenings to ensure common understanding of inclusion criteria. All the above work was done by two independent researchers and was approved by third reviewers.

### Data collection, risk of bias assessment and analysis

Independent researchers evaluated the quality of the literature and were approved by third reviewers. Evaluation aspects included whether: (A)random sequences were properly generated; (B) the distributions of hidden were properly used; (C) subjects and intervention providers were properly blinded; (D) evaluators of the results were properly blinded; (E) the completeness of outcome data was properly maintained; (F) selective reporting was properly conducted (assessed by comparing outcomes specified in the methodology compared to those reported in the results section.); (G) other biases were properly disposed. According to the above specific evaluation criteria, the included studies were categorized as ‘low risk’, ‘high risk’ or ‘unclear risk’. Any disagreements were discussed and resolved with Professor Wang.

### Statistical data analysis

We used the Review Manager 5.4 software to perform the data analysis. The effects of probiotics on selected parameters were mostly analyzed using mean difference (MD) with standard deviation (SD). When the study’s authors did not provide SDs of mean differences, we calculated the SDs of outcomes using the following formula: SD² change = SD² baseline + SD² final – (2*correlation coefficient*SD baseline*SD final), assuming that the correlation coefficient is 0.5 [17]. A *P* ≤ 0.05 was considered statistically significant. To investigate statistical heterogeneity, we visually assessed the forest plots and examined the heterogeneity. Heterogeneity between studies was analyzed using a chi-square test. When I² was >50% or *P* < 0.1, high heterogeneity was indicated, and random effects model was used; otherwise, the fixed effects model was used.

## Results

### Literature retrieval, research characteristics, and methodological quality assessment

**Fig. 1:**
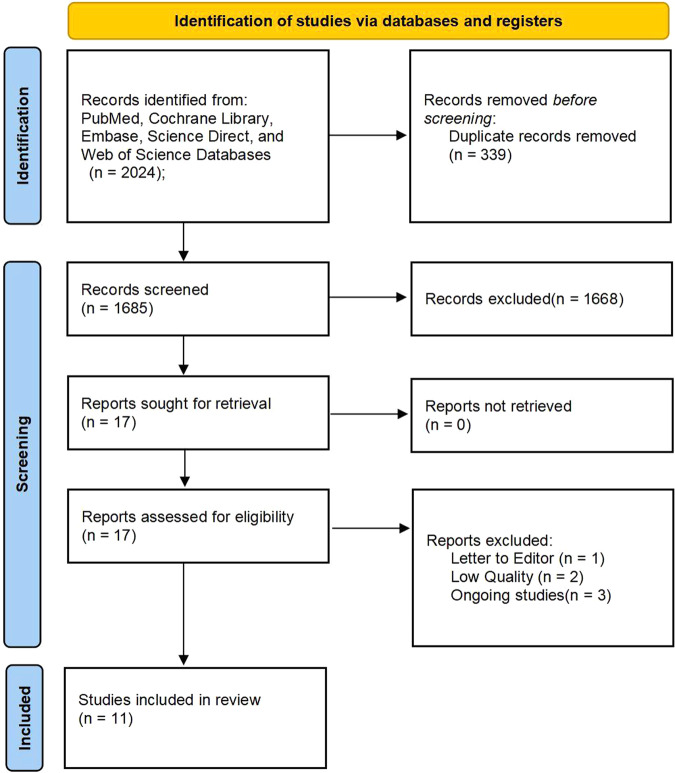
PRISMA flow diagram of the study selection process. A total of 2024 documents were included based on the search strategy and finally 11 studies were included in the analysis for detailed assessment.

**Fig. 2:**
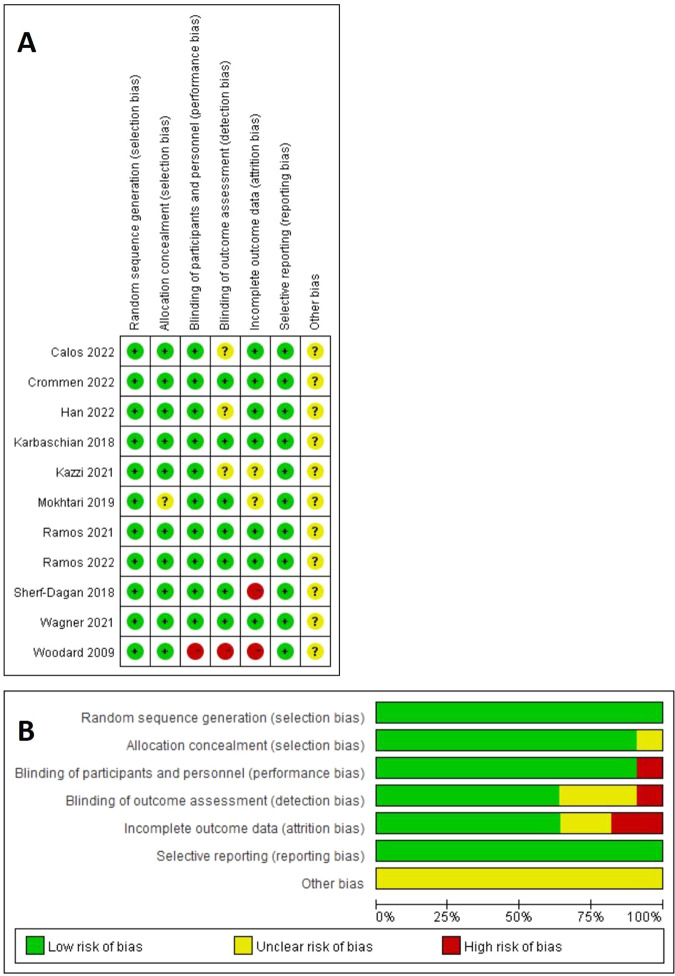
Quality assessments of the included RCTs articles. **A** Risk of bias graph. **B** Risk of bias summary for all RCT studies.

A total of 2024 documents were included based on the search strategy. Finally, 11 studies [18–28] between 2009 and 2022, including 559 patients (279 patients in the probiotic group and 280 patients in the placebo group) were included in the analysis by assessing the full text of articles eligible for detailed assessment. The study of Chen [29] and Fernandes [30] et al. tended to meet the inclusion criteria, but they were excluded for the subjects were small and low quality. Sample sizes ranged from 29 [20] to 80 [22]. The process diagram was shown in Fig. 1. The characteristics of all included RCTs [18–28] were summarized in Table 1, with their methodological quality highlighted in Fig. 2. The durations of the probiotics were 3 months after bariatric surgery except the studies of Woodard and Han et al. The studies of Karbaschian and Mokhtari et al. were from 4 weeks before surgery to 12 weeks after bariatric surgery. Adequate random sequence generation was reported in 11 trials. Allocation concealment was reported in 10 trials. Adequate blinding of participants and personnel for objective outcomes was achieved in 10 of 11 trials. Adequate blinding of outcome assessments was achieved in 7 trials. Attrition bias was reported in 5 trials and reporting bias was not reported. Study protocols were registered in 5 [21, 22, 24, 25, 28] of 11 trials. The study by Han et al. [21] comprised 3 groups: symbiotic, prebiotic, and placebo; thus, in accordance with our methods, we only included prebiotic and placebo groups.

**Table 1:** Characteristics of the included randomized controlled trials in systematic review.

Study(Author,Year)	Procedure	Size(I/N)	Intervetion(Dose and type)	Control	Duration	Analyzed outcomes included
Woodard, 2009	RNYGB	22/22	1 capsule/d Each capsule contains 2.4 billion live cells of *Lactobacillus*.	No supplementation	6 M	%EWL, Vitamin B_12_.
Karbaschian, 2018	OAGB	23/23	Daily Seven species of probiotic bacteria(*Lactobacillus casei* (3.5*10^9^ CFU/g), *Lactobacillus rhamnosus*(7.5*10^8^ CFU/g), *Streptococcus thermophilus* (1*10^8^ CFU/g),*Bifidobacterium breve*(1*10^10^ CFU/g), *Lactobacillus acidophilus* (1*10^9^ CFU/g), *Bifidobacterium longum*(3.5*10^9^ CFU/g),and *Lactobacillus bulgaricus* (1*10^8^ CFU/g)) and 38.5 mg fructo-oligosaccharide.	Daily Placebo	4 M(From 4 weeks before surgery to 12 weeks after bariatric surgery)	Plasma Glucose, Insulin, HOMA-IR, QUICKI, TC, TG, HDL, LDL, BMI, Weight, WC, Vitamin B_12_, 25-OH Vitamin D_3_, Folate.
Sherf-Dagan, 2018	LSG	40/40	2 capsules/d Each capsule contains 11 different species including 25 billion active bacteria (*Lacidophilus*, *L. rhamnosus*, *L. casei, L. paracasei*, *Lactobacillus plantarum*, *Lactococcus lactis*, *Bifidobacterium bifidum*, *B. breve*, *B. longum*, *Bifidobacterium infatis*, *S.thermophiles*).	2 capsules/d Placebo	3 M(The change of IL-6 and TNF-a were measured after 6 months)	ALT, AST, GGT, Plasma Glucose, HbA1c, HOMA-IR, TC, TG, HDL, LDL, IL-6(6 M), TNF-α(6 M), CRP,%EWL, BMI, WC, Ferritin, Hb.
Mokhtari, 2019	OAGB	23/23	1 capsule/d Each capsule contains seven species of probiotic bacteria(*Lactobacillus casei* (3.5*10^9^ CFU/g), *Lactobacillus rhamnosus*(7.5*10^8^ CFU/g), *Streptococcus thermophilus* (1*10^8^ CFU/g),*Bifidobacterium breve*(1*10^10^ CFU/g), *Lactobacillus acidophilu*s (1*10^9^ CFU/g), *Bifidobacterium longum*(3.5*10^9^ CFU/g),and *Lactobacillus bulgaricus* (1*10^8^ CFU/g)) and 38.5 mg fructo-oligosaccharide.	1 capsule/d Placebo	4 M(From 4 weeks before surgery to 12 weeks after bariatric surgery)	IL-6, TNF-α, CRP,%EWL, Dietary Energy, Dietary Protein, Dietary Cholesterol, Dietary Carbohydrate, Dietary Fat, Dietary Fiber.
Kazzi, 2021	LSG	15/20	1 capsule/d Each capsule contains 300 mg of combined *Bacillus* coagulans and galactomannans constituting 4.5billion live cells.	1 capsule/d Placebo	3 M	ALT, AST, Insulin, HbA1c, TC, TG, HDL, LDL, CRP,%EWL, BMI, Vitamin B_12_.
Wagner, 2021	RYGB	34/39	2 tablets/d Each tablet contains 5 billion *Lactobacillus acidophilus*, *Bifidobacterium lactis*.	2 tablets/d Tablets(starch)	3 M	%EWL, Dietary Energy, Dietary Protein, Dietary Fat, Dietary Carbohydrates, Dietary Fibers, GSRS.
Ramos, 2021	RYGB	38/33	1 tablet/d Each tablet contains 5 billion *Lactobacillus acidophilus*, *Bifidobacterium lactis*.	1 tablet/d Placebo	3 M	Plasma Glucose, Serum Insulin, HbA1c, HOMA-IR, QUICKI, TC, TG, HDL, LDL,%EWL, BMI, WC, Vitamin B_12_,25-OH Vitamin D_3_, Serum Folate.
Carlos, 2022	RYGB	37/32	1 tablet/d Each tablet contains 5 billion *Lactobacillus acidophilus* and *Bifidobacterium lactis*.	1 tablet/d Placebo	3 M	%EWL, Weight, BES, YFBS.
Crommen, 2022	MGB	25/23	Daily A multistrain mixture containing *Lactobacillus acidophilus Bifidobacterium breve,B.longum,L. delbrueckii susp. bulgaricus, L. belveticus,L. plantarum, L. rhamnosus, L. casei, Lactococcus lactissusp.lactis*, and *Streptococcus thermophiles*(15*10^9^ CFU/4 g),and 3.9 g specific micronutrient mixture.	Daily Micronutrient mixture and a placebo powder	3 M	ALT, AST, GGT, Plasma Glucose, serum insulin, HbA1c, HOMA-IR, TC, TG, LDL, HDL, IL-6, CRP,%EWL, BMI, Weight, WC, Ferritin, Hb.
Han, 2022	SG/OAGB	33/31	Twice/d 1 g *Clostridium butyricum* MIYAIRI, 5 billion colony-forming units.	Twice/d Digestive enzyme	6 M	Adverse effects.
Ramos, 2022	RYGB	13/16	2 tablets/d 5 billion *Lactobacillus acidophilus* and 5 billion *Bifidobacterium lactis*.	2 tablet/d Placebo	3 M	%EWL, BMI, Weight.

*I/N* Intervention/control, *ALT* Alanine Aminotransferase, *AST* Aspartate Aminotransferase, *GGT* Glutamyl Transpeptidase, *HbA1c* Hemoglobin A1c, *HOMA-IR* Homeostatic Model Assessment for Insulin Resistance, *QUICKI* Quantitative Insulin Sensitivity Check Index, *TC* Total Cholesterol, *TG* Triglyceride, *HDL* High Dentity Lipid, *LDL* Low Dentity Lipid, *IL-6* Interleukin-6, *TNF-a* Tumor Necrosis Factor-a, *CRP* C-reactive protein, *%EWL* excess weight loss, *BMI* body mass index, *WC* Waist Circumference, *Hb* hemoglobin, *BES* Binge Eating Score, *YFBS* The Yale Food Addiction Scale, *GSRS* Gastric Symptom Rating Scale.

### Effects of probiotics on liver function levels

**Fig. 3:**
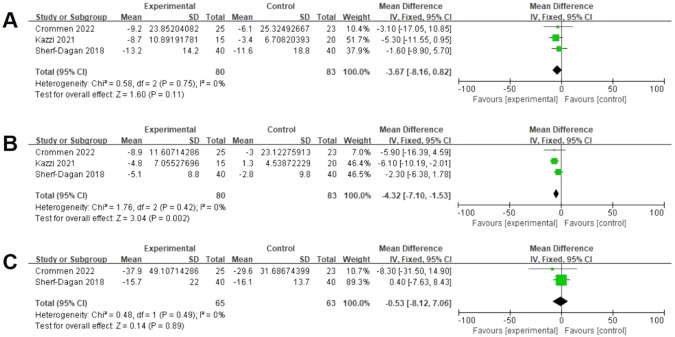
Effects of probiotics on biomarkers of the liver function levels. **A** Three studies reported data on ALT and there was no significant difference between both groups ALT. **B** Three studies reported data on AST and there was a significant difference between both groups AST. **C** Two studies reported data on GGT and there was no significant difference between both groups.

Three studies assessed data on ALT at the end of the third month (The following descriptions, unless otherwise stated, indicate that the results were measured three months after bariatric surgery) postoperatively [18, 22, 28]. There was no significant difference between both groups (80 vs 83, MD = −3.67 U/L; 95% CI [−8.16, 0.82], *p* = 0.11), with no significant heterogeneity (I² = 0%, *P* = 0.75) (Fig. 3A). Three studies reported data on AST postoperatively [18, 22, 28]. There was a significant difference between both groups (80 vs 83, MD = −4.32 U/L; 95% CI [−7.10, −1.53], *p* = 0.002), with no significant heterogeneity (I² = 0%, *P* = 0.42) (Fig. 3B). Two studies reported data on GGT postoperatively [22, 28]. There was no significant difference between both groups (65 vs 63, MD = −0.53 U/L; 95% CI [−8.12, 7.06], *p* = 0.89), with no significant heterogeneity (I² = 0%, *P* = 0.49) (Fig. 3C).

### Effects of probiotics on glycemic parameters

**Fig. 4:**
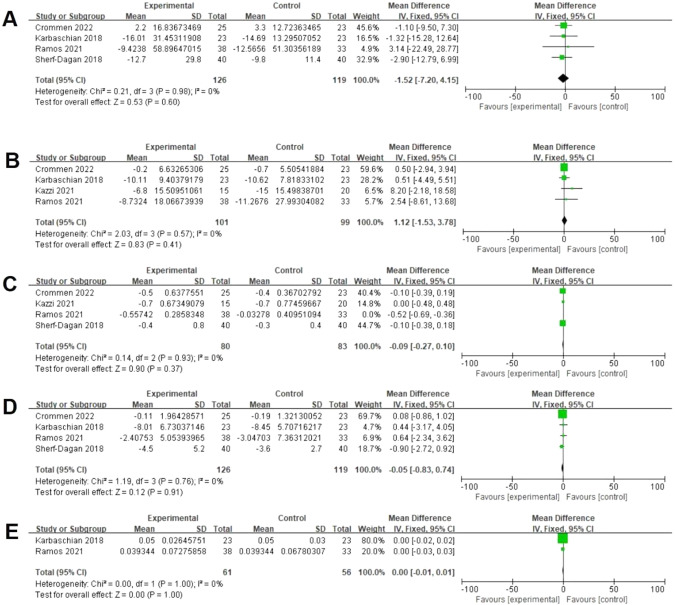
Effects of probiotics on glycemic parameters. **A** Four studies reported data on plasma glucose and there was no significant difference between both groups. **B** Four studies reported data on insulin and there was no significant difference between both groups. **C** Four studies reported data on HbA1c and there was no significant difference between both groups. **D** Four studies reported data on HOMA-IR and there was no significant difference between both groups. **E** Two studies reported data on QUICKI and there was no significant difference between both groups.

Four studies assessed changes in blood glucose from baseline [18, 22, 24, 28]. There was no significant difference between both groups (126 vs 119, MD = −1.52 mg/dL; 95% CI [−7.20, 4.15], *p* = 0.60), with no significant heterogeneity (I² = 0%, *P* = 0.98) (Fig. 4A). Four studies reported data in insulin from postoperatively [18, 19, 24, 28]. There was no significant difference between both groups (101 vs 99, MD = 1.12 mU/L; 95% CI [−1.53, 3.78], *p* = 0.41), with no significant heterogeneity (I² = 0%, *P* = 0.57) (Fig. 4B). Four studies reported data on HbA1c postoperatively [18, 19, 22, 28]. There was no significant difference between both groups (80 vs 83, MD = −0.09%; 95% CI [−0.27, 0.10], *p* = 0.37), with a no significant heterogeneity (I² = 0%, *P* = 0.93). (Fig. 4C). Four studies assessed data on HOMA-IR postoperatively [18, 22, 24, 28]. There was no significant difference between both Groups (126 vs 119, MD = −0.05; 95% CI [−0.83, 0.74], *p* = 0.91), with no significant heterogeneity (I² = 0%, *P* = 0.76) (Fig. 4D). Two studies assessed data on QUICKI postoperatively [18, 24]. There was no significant difference between both groups (61 vs 56, MD = 0.00; 95% CI [−0.01, 0.01], *p* = 1.00), with no significant heterogeneity (I² = 0%, *P* = 1.00) (Fig. 4E).

### Effects of probiotics on blood lipid levels

**Fig. 5:**
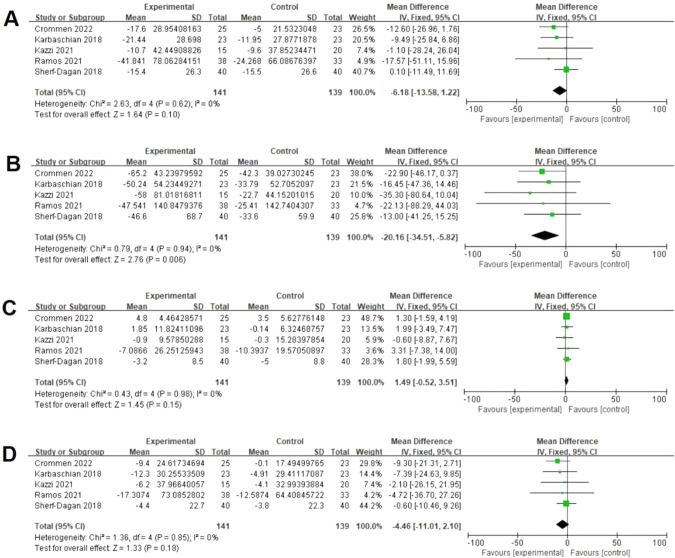
Effects of probiotics on blood lipid levels. **A** Five studies reported data on TC and there was no significant difference between both groups. **B** Five studies reported data on TG and there was a significant difference between both groups. **C** Five studies reported data on HDL and there was no significant difference between both groups. **D** Five studies reported data on LDL and there was no significant difference between both groups.

Five studies assessed data on TC from baseline postoperatively [18, 19, 22, 24, 28]. There was no significant difference between both groups (141 vs 139, MD = −6.18 mg/dL; 95% CI [−13.58, 1.22], *p* = 0.10), with no significant heterogeneity (I² = 0%, *P* = 0.62) (Fig. 5A). Five studies assessed data on TG from baseline postoperatively [18, 19, 22, 24, 28]. There was a significant difference between both groups (141 vs 139, MD = −20.16 mg/dL; 95% CI [−34.51, −5.82], *p* = 0.006), with no significant heterogeneity (I² = 0%, *P* = 0.94) (Fig. 5B). Five studies assessed data on HDL-C from baseline postoperatively [18, 19, 22, 24, 28]. There was no significant difference between both groups (141 vs 139, MD = 1.49 mg/dL; 95% CI [−0.52, 3.51], *p* = 0.15), with no significant heterogeneity (I² = 0%, *P* = 0.98) (Fig. 5C). Five studies assessed data on LDL from baseline postoperatively [18, 19, 22, 24, 28]. There was no significant difference between both groups (141 vs 139, MD = −4.46 mg/dL; 95% CI [−11.01, 2.10], *p* = 0.18), with no significant heterogeneity (I² = 0%, *P* = 0.85) (Fig. 5D).

### Effects of probiotics on inflammatory factors levels

Three studies assessed data on IL-6 from baseline postoperatively [22, 25, 28] (The result of Sherf-Dagan included is 6 months postoperatively). There was no significant difference between both groups (88 vs 86, MD = 0.24 pg/mL; 95% CI [−0.75, 1.23], *p* = 0.64), with no significant heterogeneity (I² = 0%, *P* = 0.38) (Table 2). Two studies assessed data on TNF-α from baseline [22, 24] (The result of Sherf-Dagan included is 6 months postoperatively). There was no significant difference between both groups (63 vs 63, MD = −5.64 pg/mL; 95% CI [−15.78, 4.49], *p* = 0.28), with a high level of heterogeneity (I² = 87%, *P* = 0.005) (Table 2). Four studies assessed data on CRP from baseline postoperatively [19, 22, 24, 28]. There was no significant difference between both groups (103 vs 106, MD = 1.40 mg/L; 95% CI [−1.33, 4.13], *p* = 0.32), with a significant heterogeneity (I² = 58%, *P* = 0.07) (Table 2).

**Table 2:** Summary of evidence findings table.

Outcome	No.of studies(I/N)	Effect size (MD,95% CI)	*P* value	I²
IL-6	3 (88/86)	0.24 pg/mL, [−0.75, 1.23]	0.64	0%
TNF-a	2 (63/63)	−5.64 pg/mL, [−15.78, 4.49]	0.28	87%
CRP	4 (103/106)	1.40 mg/L, [−1.33, 4.13]	0.32	58%
Folate	2 (61/56)	0.48 nmol/L, [−0.98,1.93]	0.52	0%
Ferritin	2 (57/62)	−14.50 ng/mL, [−39.59, 10.58]	0.26	0%
Hb	2 (57/62)	−0.11 g/dL, [−0.40, 0.18]	0.45	18%
GSRS	1 (34/39)	−0.34, [−0.46, −0.22]	<0.00001	N/A
BES(3 M)	1 (37/32)	−1.12, [−1.93, −0.31]	0.007	N/A
BES(12 M)	1 (22/22)	−1.40, [−2.40, −0.40]	0.006	N/A
YFAS(3 M)	1 (37/32)	−3.29, [−6.86, 0.28]	0.07	N/A
YFAS(12 M)	1 (22/22)	−5.06, [−9.51, −0.61]	0.03	N/A
Defection	1 (40/40)	−0.50, [−0.92, −0.08]	0.02	N/A
Adverse events	1 (33/31)	0.39, [0.09, 1.65]	0.2	N/A

*MD* Mean difference, *CI* Confidence interval, *Hb* Hemoglobin, *BES* Binge Eating Scale, *YFAS* The Yale Food Addition Scale.

### Effects of probiotics on general measure

**Fig. 6:**
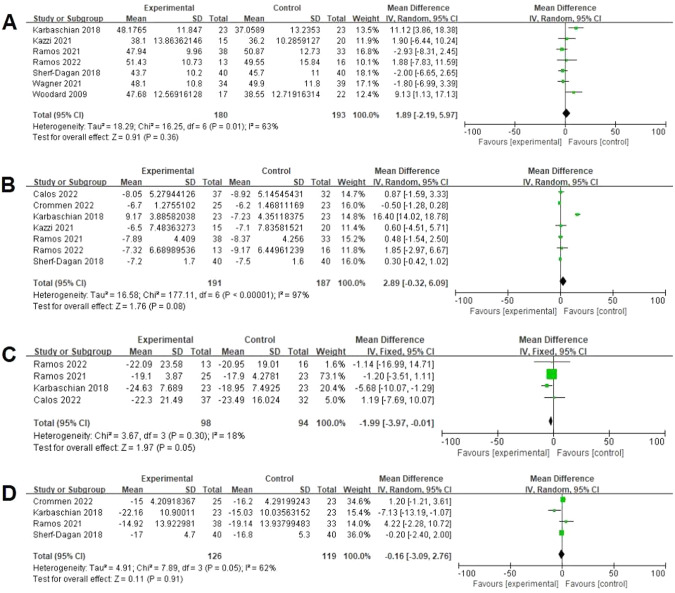
Effects of probiotics on general measure. **A** Seven studies reported data on %EWL and there was no significant difference between both groups. **B** Seven studies reported data on BMI and there was no significant difference between both groups. **C** Four studies reported data on weight and there was a significant difference between both groups. **D** Four studies reported data on WC and there was no significant difference between both groups.

Meta-analysis of seven studies did not indicate a significant effect of probiotics supplementation on %EWL (180 vs 193, MD = 1.89%; 95% CI [−2.19, 5.97], *p* = 0.36) [18–20, 22–24, 27], with a significant heterogeneity (I² = 63%, *P* = 0.01) (Fig. 6A). Seven studies assessed data on BMI from baseline postoperatively [18–20, 22, 24, 26, 28]. There was a significant difference between both groups (191 vs 187, MD = −2.89 kg/m²; 95% CI [−0.32, 6.09], *p* = 0.08), with a significant heterogeneity (I² = 97%, *P* < 0.00001) (Fig. 6B). Four studies assessed data in weight from baseline postoperatively [20, 24, 26, 28]. There was a significant difference between both groups (98 vs 94, MD = −1.99 kg; 95% CI [−3.97, −0.01], *p* = 0.05), with no significant heterogeneity (I² = 18%, *P* = 0.30) (Fig. 6C). Four studies assessed data in WC from baseline postoperatively [18, 22, 24, 28]. There was no significant difference between both groups (126 vs 119, MD = −0.16 cm; 95% CI [−3.08, 2.76], *p* = 0.91), with no significant heterogeneity (I² = 62%, *P* = 0.05) (Fig. 6D).

### Effects of probiotics on Vitamin B_12_, 25-hydroxy Vitamin D_3_, folate, ferritin and Hb

**Fig. 7:**
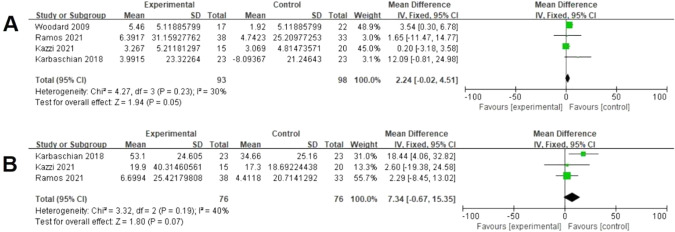
Effects of probiotics on vitamin B and 25-hydroxy vitamin D_3_. **A** Four studies reported data on vitamin B_12_ and there was a significant difference between both groups. **B** Three studies reported data on 25-hydroxy vitamin D_3_ and there was no significant difference between both groups.

Four studies assessed data on serum vitamin B_12_ from baseline postoperatively [18, 19, 23, 24] (The result of Woodard et al. included is 6 months postoperatively.). There was a significant difference between both groups (93 vs 98, MD = 2.24 pg/dL; 95% CI [−0.02, 4.51], *p* = 0.05), with no significant heterogeneity (I² = 30%, *P* = 0.23) (Fig. 7A). Three studies assessed data on serum 25-hydroxy vitamin D_3_ from baseline postoperatively [18, 19, 24]. There was no significant difference between both groups (76 vs 76, MD = 7.34 mg/dL; 95% CI [−0.67, 15.35], *p* = 0.07), with no significant heterogeneity (I² = 40%, *P* = 0.19) (Fig. 7B). Two studies assessed data on serum folate from baseline postoperatively [18, 24]. There was no significant difference between both groups (61 vs 56, MD = 0.48 nmol/L; 95% CI [−0.98, 1.93], *p* = 0.52), with no significant heterogeneity (I² = 0%, *P* = 0.63) (Table 2). Two studies assessed data on serum ferritin from baseline postoperatively [22, 28]. There was no significant difference between both groups (57 vs 62, MD = −14.5 ng/mL; 95% CI [−39.59, 10.58], *p* = 0.26), with no significant heterogeneity (I² = 0%, *P* = 0.83) (Table 2). Two studies assessed data on hemoglobin (Hb) from baseline postoperatively [22, 28]. There was no significant difference between both groups (57 vs 62, MD = −0.11 g/dL; 95% CI [−0.40, 0.18], *p* = 0.45), with no significant heterogeneity (I² = 18%, *P* = 0.27) (Table 2).

### Effects of probiotics on dietary energy, protein, cholesterol, carbohydrate, fat, fiber

**Fig. 8:**
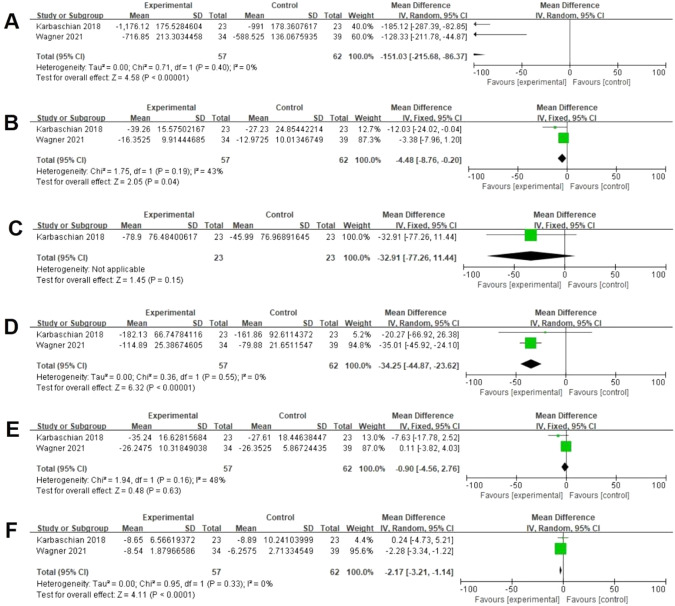
Effects of probiotics on dietary energy, protein, cholesterol, carbohydrate, fat, and fiber. **A** Two studies reported data on dietary energy and there was a significant difference between both groups. **B** Two studies reported data on dietary protein and there was a significant difference between both groups. **C** One study reported data on dietary cholesterol and there was no significant difference between both groups. **D** Two studies reported data on dietary carbohydrate and there was a significant difference between both groups. **E** Two studies reported data on dietary fat and there was no significant difference between both groups. **F** Two studies reported data on dietary fiber and there was a significant difference between both groups.

Two studies assessed data on dietary energy postoperatively [24, 27]. There was a significant difference between both groups (57 vs 62, MD = −151.03 kcal, 95% CI [−215.68, −86.37], *p* < 0.00001), with no significant heterogeneity (I² = 0%, *P* = 0.40) (Fig. 8A). Two studies assessed data on dietary protein postoperatively [24, 27]. There was a significant difference between both groups (57 vs 62, MD = −4.48 g/day, 95% CI [−8.76, −0.20], *p* = 0.04), with no significant heterogeneity (I² = 43%, *P* = 0.19) (Fig. 8B). One study assessed data on dietary cholesterol postoperatively [24]. There was no significant difference between both groups (23 vs 23, MD = −32.91 mg/day, 95% CI [−77.26, 11.44], *p* = 0.15) (Fig. 8C). Two studies assessed data on dietary carbohydrate postoperatively [24, 27]. There was a significant difference between both groups (57 vs 62, MD = −34.25 g/day, 95% CI [−44.87, −23.62], *p* < 0.00001), with no significant heterogeneity (I² = 0%, *P* = 0.55) (Fig. 8D). Two studies assessed data on dietary fat postoperatively [24, 27]. There was no significant difference between both groups (57 vs 62, MD = −0.90 g/day, 95% CI [−4.56, 2.76], *p* = 0.63), with no significant heterogeneity (I² = 48%, *P* = 0.16) (Fig. 8E). Two studies assessed data on dietary fiber postoperatively [24, 27]. There was a significant difference between both groups (57 vs 62, MD = −2.17 g/day, 95% CI [−3.21, −1.14], *p* < 0.0001), with no significant heterogeneity (I² = 0%, *P* = 0.33) (Fig. 8F).

### Effects of probiotics on gastrointestinal symptoms scores, BES, YFBS

There was a significant difference between both groups in the evolutions of GSRS [27] (34 vs 39, MD = 0.34, 95% CI [−0.46, −0.22], *p* < 0.00001) (Table 2). Calos ’s study [26] showed that after give probiotics supplementation to patients with morbid obesity undergoing bariatric surgery 3 months and 1 year later, the BES and the YFAS were lower in the probiotics groups (BES (3 M): 37 vs 32, MD = −1.12, 95% CI [−1.93, −0.31], *p* = 0.007; BES (12 M): 22 vs 22, MD = −1.40, 95% CI [−2.40, −0.40], *p* = 0.006; YFAS (3 M): 37 vs 32, MD = −3.29, 95% CI [−6.86, −0.28], *p* = 0.07; YFAS (12 M): 22 vs 22, MD = −5.06, 95% CI [−9.51, −0.61], *p* = 0.03) (Table 2).

### Adverse events

No studies reported severe adverse events. Adverse events were reported in 5 studies [21–23, 27, 30]. In Han’s study [21], one patient suffered from nausea or vomiting and two patients suffered from diarrhea in probiotic group (*n* = 41). In the digestive enzyme group (*n* = 42), five patients suffered from nausea or vomiting, one patient suffered from diarrhea and one suffered from constipation (33 vs 31, MD = 0.39, 95% [0.09, 1.65], *p* = 0.2) (Table 2). In 4 studies, no adverse events associated with the interventions were observed [22, 23, 27, 30].

## Discussion

This systematic review, assessing the effects of oral probiotics supplementation in patients with morbid obesity undergoing bariatric surgery, included 11 randomized clinical trials encompassing 559 participants. The results suggested that it was statistically significant of probiotics in reducing AST, TG, weight, food intake and vitamin B_12_, which may help inform clinical physicians and patients concerning the use of probiotics.

Different probiotics had different effects on the results under different intervention durations and intervention doses. Regretfully, we were not able to select the most effective dose of probiotics by comparing our present data. However, from the analysis, we proposed that the probiotics supplementation, especially *Lactobacillus* and *Bifidobacterium*, may be beneficial in the patients with morbid obesity undergoing bariatric surgery, for 9 studies used either *Lactobacillus* or *Bifidobacterium* except the study of Kazzi and Han et al. And the durations of probiotics supplementation mostly were 3 months postoperatively except the duration of Han and Woodard et al. were 6 months (Results related with serum TNF-a and IL-6 levels in Sherf-Dagan were also 6 months) [31, 32].

We found that probiotics consumption for 3 months decreased serum AST in patients with morbid obesity undergoing bariatric surgery. NAFLD is one of the complications associated with obesity [33], and is characterized by hepatic lipid accumulation, lipotoxicity, insulin resistance, gut dysbiosis and inflammation [34]. An association between probiotics and NAFLD had been reported that the gut-liver axis was established by the portal vein which enabled direct transport of gut-derived products to the liver [35] and various metabolites produced by the gut microbiota may impact the liver and thus modulate the susceptibility of NAFLD [36, 37]. Probiotics may improve liver function through various mechanisms such as modification of the gut microbiota, reducing appetite, intestinal permeability, and modulating the immune system [11, 38–40]. Probiotics had a significant effect on the function of the mucosal immune systems [41], modulating different signaling pathways involved in inflammatory and antioxidant processes, thus providing therapeutic effects [42, 43]. Probiotics were considered as a novel strategy for the management of NAFLD [44]. In our meta-analysis, we found that probiotics consumption for 3 months significantly reduced AST levels in patients with morbid obesity who underwent bariatric surgery. In the study of Kazzi et al., the probiotics were *Bacillus*. It was reported in the study of Salem et al. that the released exopolysaccharide by *Bacillus* alleviated CCl_4_ -induced liver injury in mice by lowering the activities of AST levels [45]. In the study of Crommen and Sherf-Dagan et al., the bacterium types included *Lactobacillus* and *Bifidobacterium*. It was reported *Lactobacillus* and *Bifidobacterium* can protect against NAFLD, and restored liver functions to normal levels [46, 47]. This suggested that probiotics may be used as an adjunct therapy for patients with morbid obesity undergoing bariatric surgery so to help in alleviating liver damage in NAFLD.

We also found that probiotics consumption for 3 months resulted in a significant decrease in serum TG level. In the studies included, the bacterium types included in probiotics were *Lactobacillus* and *Bifidobacterium* except the probiotics type of Kazzi et al. was *Bacillus*. Obesity was considered one of the common secondary causes of hyperlipidemia [48]. Bariatric surgery can improve intestinal flora, and probiotics may improve intestinal flora on the basis of bariatric surgery [27, 49]. Altered intestinal flora can affect lipid metabolism in various mechanisms. In the study of Aziz et al., it was reported that *Lactobacillus* restored lipolytic gene expression [46]. *Lactobacillus* and *Bifidobacterium* can improve the short-chain fatty acids (SCFAs) which had been shown to improve lipid signaling [50, 51]. *Lactobacillus* may affect TC levels by secreting biological inhibitors, such as cholesterol lipid coenzyme A inhibitors, to inhibit the formation of key enzymes or precursors in the cholesterol biosynthesis pathway [52]. *Bacillus* may alter bile acid composition and alleviate high-carbohydrate diet-induced hepatic lipid accumulation [53]. After related literature searches, we found that there were relatively fewer studies on how probiotics affected serum TG, LDL and HDL compared to TC, thus more in-depth studies could be conducted in this area in the future.

We also found that probiotics consumption for 3 months resulted in a significant decrease in weight. Probiotics modulated microbiota in a way to increase bile salt hydrolase activity, which in turn increased taurine abundance in the gut that stimulated tight junctions and suppressed gut leakiness [54], thus reducing inflammation [55]. The reduction in inflammation led to increased concentrations of leptin, glucagon-like peptide 1, and pancreatic polypeptide in the intestine, which leads to a reduction in food intake due to an increase in satiety [56–58]. A recent study reported *Fusimonas intestini*, highly colonized in humans with obesity and hyperglycemia, can produce long-chain fatty acids and facilitate diet-induced obesity consequently [59]. The study of Liang et al. and Karl et al. reported that *Lactobacillus* [60] and *Bifidobacterium* [61] can promote SCFAs which may increase energy expenditure through induction of thermogenesis in brown adipose tissue as well as browning of the white adipose tissue [62], contributing to the weight loss. In our meta-analysis, the studies of weight all included *Lactobacillus* and *Bifidobacterium*. Therefore, we proposed that the probiotics supplementation, especially *Lactobacillus and Bifidobacterium* may alter the component of intestinal microbiome and alter the SCFAs levels and finally contribute to the opposite direction of weight change. There were also studies showing that the intake of probiotics could lead to significant weight reductions, either maintaining habitual lifestyle habits or in combination with energy restriction and/or increased physical activity for an average of 12 weeks and specific strains belonging to the genus *Lactobacillus* and *Bifidobacterium* were the most used and showed the best results in reducing body weight [63, 64]. To be more precise, *Lactobacillus* and *Bifidobacterium* probiotics could contribute to weight loss in patients with morbid obesity undergoing bariatric surgery, suggesting probiotics might be a complement for patients with morbid obesity undergoing bariatric surgery.

Meta-analysis indicated that dietary energy, protein, carbohydrate and fiber were statistically reduced in the probiotics group compared to the placebo group. In Calos ’s study, they confirmed that the BES and YFAS statistically reduced in experiment group contrasting to the placebo group [26]. The probiotics type all included *Lactobacillus* and *Bifidobacterium*. The gut-brain axis can be altered by diet [65]. Several studies reported that probiotics may affect food appetite. Favorable effects of probiotics had been shown on regulating adiponectin, leptin, secretion and desire to eat [66]. It was reported that *Lactobacillus* tended to reduce energy intake [67]. Therefore, probiotics may present an appetite altering effect, contributing to a reduction of food intake, leading to an improvement in lipids metabolism and weight loss.

Significant improvements in vitamin B_12_ was also found in our meta-analysis. The bacterium included in the meta-analysis were mainly *Lactobacillus* and *Bifidobacterium*. The bacterium in the study of Kazzi et al. was *Bacillus*. Probiotics consumption may be an appropriate strategy to improve vitamin B_12_ status through intestinal microbiota modulation. *Lactobacillus* and *Bifidobacterium* were related to vitamin B_12_ metabolite transport systems [68]. It was reported that *Lactobacillus* supplement ameliorated vitamin B_12_ deficiency as an adjunctive therapy in canine clinical practice. *Bacillus* was a bacterium that had been used in the past for the industrial production of vitamin B_12_ [69]. Thus, we proposed the probiotics, including *Lactobacillus*, *Bacillus* and *Bifidobacterium* were beneficial in improving the vitamin B_12_ levels in patients with morbid obesity undergoing bariatric surgery.

The adverse events reported in patients with morbid obesity undergoing bariatric surgery were insufficient. We could not clearly confirm whether probiotics would contribute to adverse events. But in the meta-analysis, 559 patients included, no severe adverse events were reported. After weight loss surgery, patients may experience a variety of post-operative complications, such as vomiting or diarrhea, even without the use of probiotics [70, 71]. The results of probiotics may be overshadowed by the side effects of bariatric surgery. Thus, it was not easy to determine whether the use of probiotics itself might cause side effects to the patient, or whether it might alleviate the side effects of bariatric surgery.

And this review also showed reductions in many other indicators, such as glycemic parameters, inflammatory factors levels, and an increase in serum 25-hydroxy vitamin D_3_ level, although they were not statistically significant. There were several possible reasons for these statistically insignificant effects. Firstly, the duration of probiotics supplementation was not adequate to produce an effect. Secondly, the intestinal microbiota composition in individuals with morbid obesity was not favorable to produce a significant result. Thirdly, the statistical power of this review might have been insufficient to demonstrate the effects of small change. Nevertheless, as related research progresses, the results may one day present significant. And many preclinical medical studies indicated that probiotics may affect these indicators through many mechanisms. Therefore, these indicators should be attended to in the next update. It was unclear whether probiotics could improve glycemic parameters and morbid obesity was often associated with insulin resistance [72]. In recent years, a number of preclinical studies reported a complex interplay between probiotics and the gut microbiota within the gut environment. Probiotics had antioxidant effects, which can scavenge free radicals and increase the sensitivity of tissues to insulin [73]. An altered gut environment may increase the secretion of short-chain fatty acids and thus stimulate the secretion of some glucose-lowering hormones [74, 75]. Reactive oxygen species (ROS) played vital roles in intestinal inflammation [76]. Probiotics may eliminate ROS to alleviate the oxidation and inflammation through the inhibition of NLRP3 inflammasome and increase the secretion of immunoglobulin A [77, 78]. Moreover, probiotics may also restore damaged intestinal epithelium barriers so to reduce the inflammatory factors induced by other harmful bacteria [79]. Mallard’s study supported the view that the association between obesity and lower serum 25-hydroxy vitamin D_3_ might be due to a reversed causality for an increasement of adiposity, leading to reduced concentrations of circulating 25-hydroxy vitamin D_3_ [80]. Probiotics consumption might be an appropriate strategy to improve 25-hydroxy vitamin D_3_ status through intestinal microbiota modulation [37, 81, 82]. It was reported that in animal models, probiotics prevented bone loss by regulating bone resorption in osteoclasts and bone formation by osteoblasts [82]. In humans, osteoblasts may regulate 25-hydroxy vitamin D levels and calcium absorption [81]. Besides, probiotics can improve the level of other nutrients in the body to some extent [83]. It was reported that application of special lactic acid bacteria, especially strains that produce folic acid and riboflavin as well as immune-stimulating strains, can be used as adjuvant therapy for patients suffering from various inflammatory diseases [84]. In addition, Bahareh’s results suggest that, although varying degrees of efficacy, the intake of certain probiotics in healthy subjects was associated with the status of certain other micronutrients, such as calcium, folate, iron and zinc [83, 85]. Therefore, probiotics may lead to improved nutrient absorption in patients with morbid obesity undergoing bariatric surgery and more relevant studies are needed.

This meta-analysis has several strengths. A comprehensive literature search was conducted, involving 5 electronic databases and manual searches of relevant studies. Therefore, it is unlikely that eligible studies were neglected. 6 eligible studies [19, 21, 22, 26–28] that were not included in any previous reviews [83, 86, 87] were also identified. Additionally, a pilot phase prior to data abstraction was implemented to test the extraction, thereby increasing the systematicity and accuracy of the data. In studies with more than 1 compassion or control group, only the groups including the live microbiome were included as the probiotics group. In addition, the GRADE approach was used to formally assess the quality of evidence.

Our meta-analysis also has some limitations: the number of RCTs included in the study was relatively small and bias was inevitable, so the quality of the literature was reduced. However, RCTs reduced the bias to some extent.

Despite the above-mentioned shortcomings, the reliability of this meta-analysis was strengthened by minimized incorporation of biased literature, rigorous data extraction, and strong statistical analysis by teamwork. The results of this study are still worthy of clinical reference.

## Conclusion

This study comprehensively evaluated outcome indicators associated with probiotics in the treatment of patients with morbid obesity undergoing bariatric surgery. Compared with previously published studies, we included more studies, providing a comprehensive analysis and evaluation of outcome indicators. The meta-analysis demonstrated that probiotics among patients with morbid obesity undergoing bariatric surgery had a beneficial effect on many indicators including the regulation of AST, TG, weight, food intake, and vitamin B_12_. Probiotics may be beneficial in patients with morbid obesity undergoing bariatric surgery. However, additional high-quality RCTs will be necessary for the future to further clarify the therapeutic effects of probiotics in patients with morbid obesity undergoing bariatric surgery.

## Data Availability

The data used in this publication are readily available from original source papers included in the systematic review.
